# DNA Methylation and Demethylation in Triple-Negative Breast Cancer: Associations with Clinicopathological Characteristics and the Chemotherapy Response

**DOI:** 10.3390/biomedicines13030585

**Published:** 2025-02-26

**Authors:** Kateryna Tarhonska, Mateusz Wichtowski, Thomas Wow, Agnieszka Kołacińska-Wow, Katarzyna Płoszka, Wojciech Fendler, Izabela Zawlik, Sylwia Paszek, Alina Zuchowska, Ewa Jabłońska

**Affiliations:** 1 Department of Translational Research, Nofer Institute of Occupational Medicine, St. Teresy 8 Street, 91-348 Lodz, Poland; 2 Department of Surgical Oncology, Institute of Oncology, Poznan University of Medical Sciences, Szamarzewskiego 84, 60-569 Poznan, Poland; mawichto@gmail.com; 3 Medical Practice Thomas Wow, 53 Malwowa Street, 60-175 Poznan, Poland; doctor.thomaswow@gmail.com; 4 Department of Oncological Physiotherapy, Medical University of Lodz, Paderewskiego 4, 93-509 Lodz, Poland; agnieszka.kolacinska@umed.lodz.pl; 5 Department of General, Gastroenterological and Oncological Surgery, Warsaw Medical University, Banacha 1a, 02-097 Warsaw, Poland; 6 Department of Biostatistics and Translational Medicine, Medical University of Lodz, Mazowiecka 15, 92-215 Lodz, Poland; katarzyna.ploszka@umed.lodz.pl (K.P.); wojciech.fendler@umed.lodz.pl (W.F.); 7 Department of General Genetics, Faculty of Medicine, Collegium Medicum, University of Rzeszow, 35-310 Rzeszow, Poland; izazawlik@gmail.com (I.Z.); sylwia.paszek@wp.pl (S.P.); alzuch4@gmail.com (A.Z.); 8 Department of Chemical Safety, Nofer Institute of Occupational Medicine, St. Teresy 8 Street, 91-348 Lodz, Poland; ewa.jablonska@imp.lodz.pl

**Keywords:** triple-negative breast cancer, DNA methylation, DNA demethylation, 5-methylcytosine, 5-hydroxymethylcytosine, epigenetic markers, neoadjuvant chemotherapy, Ki-67

## Abstract

**Objectives:** Triple-negative breast cancer (TNBC) is an aggressive cancer subtype with limited treatment options due to the absence of estrogen, progesterone receptors, and HER2 expression. This study examined the impact of DNA methylation and demethylation markers in tumor tissues on TNBC patients’ response to neoadjuvant chemotherapy (NACT) and analyzed the correlation between 5-methylcytosine (5-mC) and 5-hydroxymethylcytosine (5-hmC) and clinicopathological characteristics, offering new insights into the predictive value of these epigenetic markers. **Methods:** The study included 53 TNBC female patients, 19 of whom received neoadjuvant chemotherapy (NACT) before surgery. Global DNA methylation and demethylation levels were quantified using an ELISA-based method to measure 5-mC and 5-hmC content in DNA isolated from pre-treatment biopsy samples (in patients undergoing NACT) and postoperative tissues (in patients without NACT). **Results:** In patients who received NACT, those with disease progression had significantly higher pretreatment levels of 5-hmC (*p* = 0.028) and a trend toward higher 5-mC levels (*p* = 0.054) compared to those with pathological complete response, partial response, or stable disease. Higher 5-mC and 5-hmC levels were significantly associated with higher tumor grade (*p* = 0.039 and *p* = 0.017, respectively). Additionally, a positive correlation was observed between the Ki-67 proliferation marker and both 5-mC (r_S_ = 0.340, *p* = 0.049) and 5-hmC (r_S_ = 0.341, *p* = 0.048) levels in postoperative tissues. **Conclusions:** Our study highlights the potential of global DNA methylation and demethylation markers as predictors of tumor aggressiveness and chemotherapy response in TNBC. Further research in larger cohorts is necessary to validate these markers’ prognostic and predictive value.

## 1. Introduction

Breast cancer is the second most commonly diagnosed cancer worldwide, with 2.3 million new cases across both sexes. In women, it remains the most frequently diagnosed cancer, accounting for a quarter of all cases in 2022. That same year, an estimated 666,000 women were expected to die from the disease [1]. In Poland, breast cancer is the most frequently diagnosed cancer among women, with a standardized incidence rate of 102/10^5^, and it is the second leading cause of cancer-related deaths, with a standardized mortality rate of 31/10^5^ [2]. Triple-negative breast cancer (TNBC) is a particularly aggressive subtype of breast cancer that represents approximately 15–20% of all breast cancers diagnosed worldwide and is characterized by the absence of estrogen receptors (ER-), progesterone receptors (PR-), and human epidermal growth factor receptor 2 (HER2-), making it resistant to conventional hormonal therapies and targeted treatments [3]. Recent research has highlighted the critical role of epigenetic modifications, particularly DNA methylation, in the pathogenesis and progression of TNBC [4]. DNA methylation, the addition of a methyl group to the cytosine base of DNA by DNA methyltransferases (DNMTs), can lead to gene silencing and alterations in cellular behavior, contributing to the aggressive nature of TNBC. Conversely, DNA demethylation processes are equally important for understanding tumor dynamics, as they may reactivate silenced oncogenes and influence disease progression. Key molecular players in this context include 5-methylcytosine (5-mC) and its oxidized derivative, 5-hydroxymethylcytosine (5-hmC), which have been implicated in the regulation of gene expression and may serve as biomarkers for tumor behavior [5]. In TNBC, DNA methylation patterns are similar to those observed in other breast cancer subtypes, with hypermethylation typically occurring in CpG islands and shores, whereas global hypomethylation is observed in intragenic regions. Although the overall number of methylated CpG islands is similar in TNBC and non-TNBC tumors, the specific genes that are methylated differ between the different breast cancer subtypes. Epigenetic modifications have broad implications for cancer cell functions, influencing processes such as proliferation, migration, adhesion, invasion, and epithelial–mesenchymal transition (EMT), which collectively contribute to the aggressive nature of TNBC [6].

As of the latest updates from the 2023 St. Gallen Consensus, chemotherapy remains the cornerstone for treating triple-negative breast cancer due to the absence of targeted therapies. The guidelines emphasize both neoadjuvant and adjuvant chemotherapy approaches depending on the stage and characteristics of the disease.

Neoadjuvant chemotherapy (NACT) is the preferred strategy for patients with stage II and III TNBC. The recommended regimen typically includes anthracyclines and taxanes, which constitute the backbone of treatment. Adding carboplatin to the regimen has been shown to increase pathological complete response (pCR) rates, a key goal in TNBC treatment. Additionally, the integration of immune checkpoint inhibitors, such as pembrolizumab, has been a significant advance. Pembrolizumab is approved for use in combination with chemotherapy in high-risk, early-stage TNBC to improve pCR rates [7]. One of the main goals of administering neoadjuvant chemotherapy is to shrink tumors and improve surgical outcomes. Unfortunately, the efficacy of this treatment ranges from 12.7 to 64.8% [8], and understanding the underlying epigenetic landscape may provide insights into treatment responses.

Over the years, many published studies have attempted to determine which clinicopathological factors can be considered surrogates for a good response to treatment in TNBC patients. The constantly growing number of studies on the tumor microenvironment, genetic profiles and immune response provides some hope in the search for reliable target points. The identification of novel baseline biomarkers that can classify patients with TNBC as good or poor responders would be pivotal for guiding therapeutic decisions [9].

Initially, considerable faith was placed in the protein marker Ki-67 [10]. The Ki-67 index serves as an indicator of cellular proliferation. The literature contains numerous studies on TNBC, where a high Ki-67 index has been associated with an increased rate of complete pathological response (pCR) to chemotherapy [11]. However, other studies do not demonstrate this correlation [12]. A challenge in comparing these findings is caused by the lack of a universally established cutoff point for what constitutes a “high” Ki-67 level. As a result, current evidence suggests that Ki-67, as an independent marker, cannot reliably be used to predict treatment response [10].

Other proteins that have been studied for their relationship with pCR include VEGFR2, vimentin, and HAGE. VEGFR2, or vascular endothelial growth factor receptor 2, is a signaling protein involved in angiogenesis; vimentin is a cytoskeletal protein associated with mesenchymal cells; and HAGE is a helicase antigen. Elevated levels of these proteins have been correlated with a higher rate of pCR [11,13,14].

Conversely, negative correlations have been observed with proteins such as FGFR4, NUP98, E-cadherin, Bcl2, ALDH1, tumor-associated stromal clusterin, TOPK, YAP1, and MMP7, where increased levels are linked to greater tumor resistance to chemotherapy [15,16,17,18,19].

An important area of focus in predicting treatment response markers is the tumor microenvironment. In this context, tumor-infiltrating lymphocytes (TILs), particularly a high presence of CD8+ cells and a high CD8/CD4 ratio, have been associated with achieving pCR [20,21]. This is especially relevant in TNBC, where a high TIL density is correlated with improved survival outcomes [22]. Numerous studies have demonstrated a strong association between high TIL levels and a greater likelihood of achieving pCR, as well as a lower residual cancer burden (RCB) score post-treatment. The variability in cutoffs for what constitutes high or low TIL density and the differing focus on TIL density within tumor tissues versus tumor peripheries complicate the aggregation of data into large, homogeneous groups for meta-analysis [9].

A clinically significant marker is PD-L1 (programmed cell death ligand 1), which can serve as a predictive marker of the pCR rate [23]. PD-L1 expression is considered a biomarker for activated CD8+ TILs at the biological level, while at the therapeutic level, it indicates that T cells previously inhibited by PD-L1 may become reactivated following PD-L1 blockade, thereby initiating an antitumor immune response [24]. Consequently, several studies report a higher rate of patients achieving pCR when pembrolizumab is added to the standard chemotherapy regimen [25].

Studies have shown that genome-wide DNA methylation screening can effectively generate epigenetic diagnostic panels to predict the efficacy of NACT for TNBC patients [26]. By investigating how changes in global methylation and demethylation affect treatment outcomes, it can be determined whether they can serve as predictive markers for patient response to neoadjuvant chemotherapy. Importantly, investigations of DNA methylation in TNBC are scarce, as most studies have focused on more common subtypes of breast cancer, particularly ER-positive subtypes [27]. Furthermore, the specific exploration of 5-mC and 5-hmC in the context of predictive utility within TNBC appears to be very limited or not yet published.

In this study, we examined the relationship between pretreatment tumor levels of 5-mC and 5-hmC and the response to NACT in patients with TNBC. Additionally, we assessed the correlation between these markers and tumor pathological features, regardless of the treatment received.

## 2. Materials and Methods

### 2.1. Study Group and Material

The study was based on the analysis of archival, anonymized FFPE (formalin-fixed, paraffin-embedded) tumor samples, which were collected during standard hospital procedures from 53 female TNBC patients, with a median age of 57.2 years. The patients were treated at the Department of Surgical Oncology of the Provincial Multidisciplinary Centre of Oncology and Traumatology in Lodz, Poland, in the years 2017–2020. The majority of the patients (86%) were diagnosed with invasive carcinoma of no special type (NST). Nineteen patients underwent neoadjuvant chemotherapy (NACT) before surgery, whereas 34 were treated with surgery without NACT. For all patients treated with preoperative chemotherapy, the regimens used were 4× AC (doxorubicin and cyclophosphamide every 2–3 weeks) and 12× paclitaxel (every week). FFPE blocks were prepared from biopsy samples collected from the NACT group before pharmacological treatment and from tumor tissue samples collected during surgery in the group that did not receive NACT. The response to NACT was classified into four categories: pCR, partial response, stable disease, and disease progression. pCR was defined as no invasive carcinoma in the breast or axillary lymph nodes (ypT0/ypN0 TNM (tumor, nodes, metastasis) staging) after NACT. Partial response referred to a reduction in the size of the primary tumor or a decrease in the extent of disease in the axillary lymph nodes. Patients with stable disease were identified as those with no significant change in TNM stage. Disease progression was defined as an increase in the size of the primary tumor or the appearance of new lesions in axillary lymph nodes. Inclusion in a particular category was based on a comparison of tumor staging before and after treatment, using clinical and pathological parameters, respectively. The study was conducted in accordance with Declaration of Helsinki and approved by the Bioethics Committee at the Medical University of Lodz (RNN/226/11/KE). As the samples were archival and anonymized, individual informed consent was not required.

The characteristics of the patients in the study group, including clinicopathological data and response to NACT, are presented in Table 1.

### 2.2. DNA Isolation

DNA was isolated from formalin-fixed, paraffin-embedded (FFPE) tumor samples, derived from either biopsy samples or surgical samples. DNA was extracted from tissue sections via the QIAamp DNA FFPE Tissue Kit (Qiagen, Hilden, Germany) according to the manufacturer’s protocol. Initially, deparaffinization was performed using xylene. Purified tissue sections with ATL lysis buffer and proteinase K were incubated at 56 °C for 3 h until complete lysis and then at 90 °C for 1 h. Then, AL buffer and ethanol were added. The entire lysate was transferred to a QIAamp MinElute column (Qiagen, Hilden, Germany) with a silica-based membrane (included with the QIAamp DNA FFPE Tissue Kit) and washed. Finally, the DNA was eluted with ATE buffer in a volume of 50 µL. DNA concentration measurement was performed on a NanoDrop 2000c Spectrophotometer (ThermoFisher Scientific, Waltham, MA, USA). The isolated genomic DNA was stored at −20 °C until further analysis.

### 2.3. Ki-67 Staining

To assess the proliferative index, we used the mouse anti-Ki-67 antibody clone MIB-1 (Dako Denmark A/S, DK-2600 Glostrup, Denmark) with automated staining (Dako Autostainer, Dako Colorado, Inc. Fort Collins, CO 80525, USA). The nuclear reaction in tumor cells was evaluated across 10 consecutive high-power fields (HPFs, 400× magnification), and the average was calculated.

### 2.4. Global DNA Methylation and Demethylation Analysis

ELISA-based quantitation of global 5-methylcytosine (5-mC) and 5-hydroxymethylcytosine (5-hmC) levels was performed according to the protocol of Nelly N. Olova [28] with some modifications.

1. Preparation of DNA samples and standards. An accurately quantified 50 ng amount of double-stranded (ds) DNA was denatured by incubating at 98 °C for 10 min, followed by immediate cooling on ice for another 10 min. The DNA was mixed with coating buffer and ultrasensitive green fluorescent single-stranded (ss) DNA dye Quant-iT OliGreen ssDNA Reagent (Invitrogen, Waltham, MA, USA) in concentration, according to the manufacturer’s instructions. This, along with a 200 ng of DNA loading standard Lambda DNA (Thermo Scientific, Waltham, MA, USA), allows for monitoring loading precision and mathematical correction of estimated 5-mC and 5-hmC levels during the analysis step. For the 5-mC and 5-hmC standard curves, 2-fold serial dilutions of the CpGenome 5-mC and 5-hmC Human DNA Standards (Sigma–Aldrich, Burlington, MA, USA) were used, starting with 100 ng of DNA. Each DNA sample, in two replicate wells per sample, including blank wells (only coating buffer) and controls, was read fluorometrically in a Plate Reader Victor™ X3 (PerkinElmer, Waltham, MA, USA) (excitation filter 485 nm/emission filter 530 nm).
2. Passive adsorption of DNA samples and standards. The DNA was passively adsorbed onto a polystyrene black plate surface (Greiner Bio-One GmbH, Kremsmünster, Austria) for 1 h at 37 °C. The plate was then washed three times with wash buffer.
3. Blocking. To prevent nonspecific antibody binding, the plate was blocked with 100 µL of 2% BSA for 1 h at 37 °C. After blocking washing plate once with wash buffer.
4. Incubation with primary antibody. The DNA was incubated for 1 h at 37 °C with a highly specific primary antibody at a concentration of 1:1500. To analyze global 5-methylcytosine, we used the OptimAb Anti-5-Methylcytosine antibody (BI-MECY-0100, clone 33D3, Eurogentec, Seraing, Liège, Belgium). For 5-hmC, we used a 5-hydroxymethylcytosine antibody (mAb) (#39999, Active Motif, Carlsbad, CA, USA). After incubation, the plate was washed three times with wash buffer.
5. Incubation with HRP-conjugated secondary antibody. The 5-mC/5-hmC–antibody complexes were further recognized by an enzyme-conjugated secondary Goat Anti-Mouse IgG H&L (HRP) (ab205719, Abcam, Cambridge, UK) antibody at a concentration of 1:8000 to amplify the initial detection. The plate was incubated for 1 h at 37 °C.
6. Enzymatic reaction and data acquisition. A chemiluminescent substrate SuperSignal™ ELISA Femto Substrate (Thermo Scientific, Waltham, MA, USA) was finally applied to yield a measurable signal on a Multimode Plate Reader Victor™ X3 (PerkinElmer, Waltham, MA, USA), which is proportional to the amount of immobilized 5-mC/5-hmC.

Analysis. The results were analyzed via GraphPad Prism (ver. 9.0.2). First, the amount of DNA for each sample and standard was calculated via the Lambda DNA linear fit equation from the plot. The 5-mC/5-hmC content of the samples was subsequently interpolated from the linear regression standard curve (R^2^ > 0.98) and corrected with the loading DNA.

### 2.5. Statistical Analysis

Statistical analysis was conducted via STATISTICA ver. 13.3 (TIBCO Statsoftware Inc., Palo Alto, CA, USA). Differences between two groups were analyzed using Mann–Whitney U test, and for comparisons between more than two groups, Kruskal–Wallis test followed by Dunn’s test was applied. Correlations between continuous variables were evaluated using Spearman’s rank correlation coefficient. Data normality was assessed with Shapiro–Wilk test, and a *p*-value of 0.05 or lower was considered statistically significant. The correlation results are presented via scatterplots, and the difference results for the Mann–Whitney U test and Kruskal–Wallis test are presented via scatter dot plots, along with their medians and interquartile ranges. Figures were created in GraphPad Prism (ver. 7.04).

## 3. Results

The median pretreatment levels of 5-mC and 5-hmC in biopsy samples from subjects with NACT accounted for 0.176% and 0.026%, respectively, and were both significantly lower than those observed in tumor samples obtained during surgery from subjects without NACT (2.859% and 0.239%, respectively, *p* < 0.0001 for 5-mC, *p* < 0.001 for 5-mC; Table 2). The two patient groups differed significantly in terms of age (49 vs. 60 years, *p* = 0.04).

**Figure 1:**
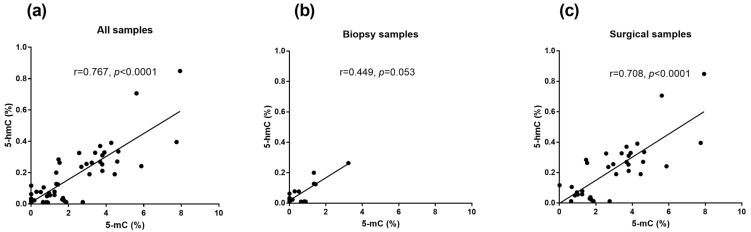
Correlations between markers of DNA methylation (5-mC) and markers of DNA demethylation (5-hmC) in the collected tumor samples, including samples from all patients (**a**), biopsies collected from patients before neoadjuvant chemotherapy (**b**) and surgical samples from patients not treated with chemotherapy (**c**).

A statistically significant positive correlation was observed between both markers in the whole group of patients (r_S_ = 0.767, *p* < 0.0001; Figure 1a). After data stratification according to the type of material, this correlation remained statistically significant only for the tumor samples obtained during surgery (r_S_ = 0.708, *p* < 0.0001; Figure 1b,c). No significant correlation was observed between 5-mC or 5-hmC and age (Figure S1).

**Figure 2:**
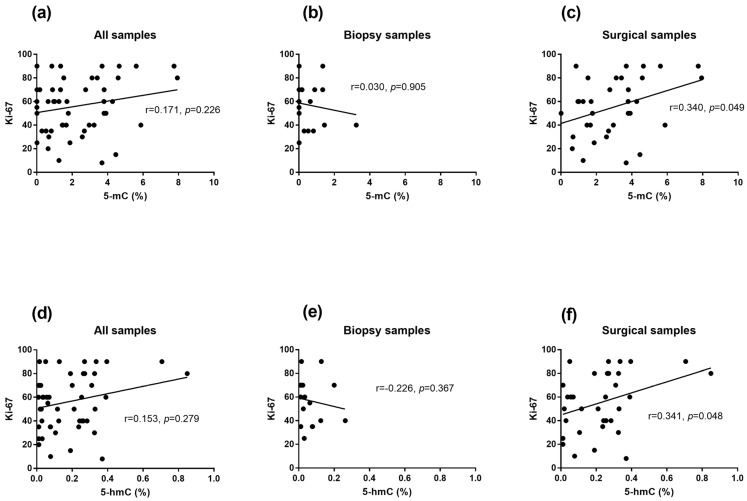
Correlation between Ki-67 and markers of DNA methylation/demethylation (5-mC/5-hmC) in the collected tumor samples, including samples from all patients (**a**,**d**), biopsies collected from patients before neoadjuvant chemotherapy (**b**,**e**) and surgical samples from patients not treated with chemotherapy (**c**,**f**).

With respect to clinicopathological characteristics, we observed a statistically significant positive correlation between 5-mC and the Ki-67 marker (r_S_ = 0.340, *p* = 0.049; Figure 2c) and between 5-hmC and the Ki-67 marker (r_S_ = 0.341, *p* = 0.048; Figure 2f) in the tumor samples obtained during surgery (patients without NACT). Similar analyses conducted in biopsies as well as in all samples together revealed no significant correlations (Figure 2a,b,d,e).

**Figure 3:**
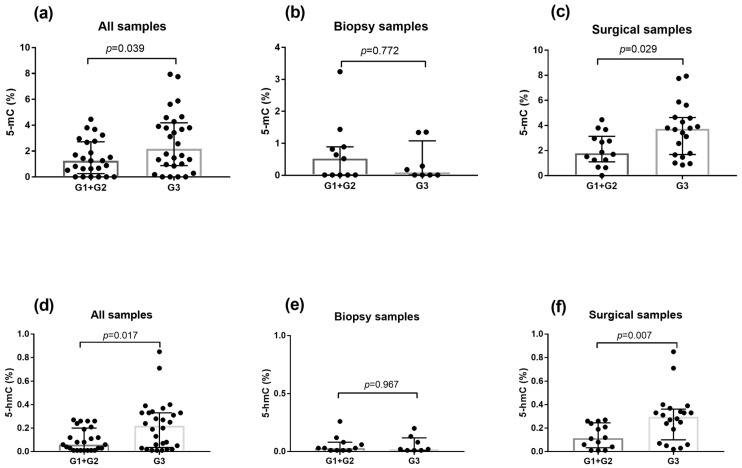
Levels of markers of DNA methylation (5-mC) and DNA demethylation (h-mC) in the collected tissue samples according to tumor grade, including samples from all TNBC patients (**a**,**d**), biopsies collected from TNBC patients before neoadjuvant chemotherapy (**b**,**e**) and surgical samples from TNBC patients not treated with neoadjuvant chemotherapy (**c**,**f**). Group differences were analyzed with the Mann–Whitney U test. Data are shown as raw values, with medians and interquartile ranges.

Both epigenetic markers were significantly associated with tumor grade (G), indicating higher levels in G3 compared to the combined G1+G2 group. Specifically, the 5-mC level was 2.173% in the G3 tumor vs. 1.251% in the combined G1+G2 group (*p* = 0.039; Figure 3a), and 5-hmC level was 0.221% in G3 tumors compared to 0.063% in the combined G1+G2 group (*p* = 0.017; Figure 3d). The G1 and G2 groups were combined because there was only one patient with G1. When the differences in marker levels according to tumor grade were examined separately in surgical and biopsy samples, a significantly greater number of markers was demonstrated between G1+G2 and G3 only in surgical samples (3.735% vs. 1.789%, *p* = 0.029 for 5-mC; 0.297% vs. 0.111%, *p* = 0.007 for 5-hmC; Figure 3b,c,e,f). No significant differences were detected between the levels of methylation markers and tumor stage (T, N) (Figures S2 and S3), with the exception of a significant difference in the level of 5-hmC in surgical samples between pT1 and pT2 (Figure S3c).

Among patients who received NACT, those who experienced disease progression during chemotherapy had the highest pretreatment levels of 5-mC and 5-hmC, with median percentages of 2.290% and 0.232%, respectively, although the differences among all four groups were not statistically significant (Table 3). These levels were comparable to those found in samples collected during surgery from patients who did not receive NACT (Table 2). The lowest pretreatment levels of 5-mC were observed in subjects with either partial (0.011%) or pathological complete response (0.095%) (Table 3). Overall, the median levels of 5-mC across the four groups of subjects who received NACT tended to decrease in the order of partial response < pCR < stable disease < disease progression, although the difference was not statistically significant according to the Kruskal–Wallis test (*p* = 0.129). In the case of 5-hmC, the trend was stable disease < pCR < partial response < disease progression (*p* = 0.080) (Table 3).

**Figure 4:**
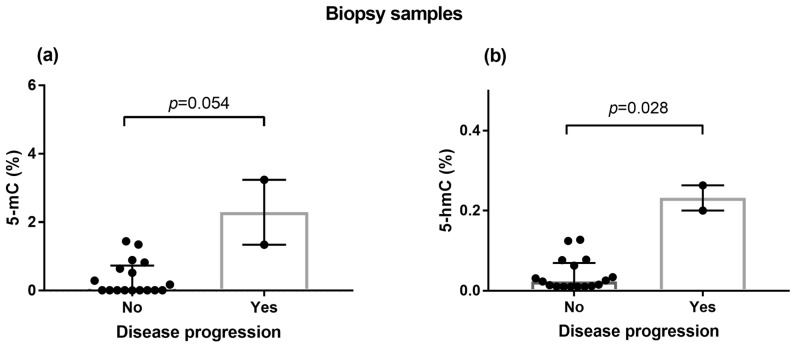
Pretreatment levels of markers of (**a**) DNA methylation (5-mC) and (**b**) DNA demethylation (h-mC) measured in biopsies collected from TNBC patients undergoing neoadjuvant chemotherapy, stratified by disease progression. The group without progression (“No”) included patients with a complete pathological response, partial response or stable disease. Group differences were analyzed with the Mann–Whitney U test. Data are shown as raw values, with medians and interquartile ranges.

Overall, patients with disease progression presented significantly higher levels of 5-hmC than did the combined group of patients with pCR, partial response, or stable disease (*p* = 0.028; Figure 4b). For 5-mC, the difference analyzed in a similar manner showed borderline significance (*p* = 0.054; Figure 4a).

## 4. Discussion

In this study, we aimed to assess the predictive potential of the global DNA methylation marker 5-mC and the demethylation marker 5-hmC in the TNBC response to NACT. These two epigenetic markers are closely related, as 5-hmC is derived from 5-mC through the action of TET (Ten-Eleven Translocation) enzymes [5]. This process establishes a dynamic equilibrium between 5-mC and 5-hmC, as evidenced in our study group by the strong positive correlation between these two epigenetic markers (r_S_ = 0.767, *p* < 0.0001). This balance reflects the activity of the DNA methylation and demethylation machinery. Notably, biopsy samples presented lower levels of 5-mC and 5-hmC than did tumor samples collected during surgery. As both types of samples were collected from two distinct patient groups and prior to any pharmacological treatment, these differences were not attributable to therapeutic effects but likely stemmed from sampling method and clinicopathological characteristics of the patients. Particularly, biopsy samples, being small and localized, may not fully capture tumor heterogeneity, unlike surgical samples, which provide a more comprehensive representation of tumor characteristics. In addition, biopsy samples were collected from larger tumors (as criterion for NACT), and these patients were generally younger than those from whom surgical samples were obtained.

In our study, we observed that among patients who underwent chemotherapy, patients who had a complete, partial, or no change response to treatment had the lowest levels of 5-mC and 5-hmC markers compared to those whose disease progressed. However, this observation should be treated with caution, as there were only two patients in the group with progression. Nevertheless, this is an interesting finding, which deserves further discussion. Global hypomethylation can lead to the activation of both tumor suppressor genes and oncogenes via epigenetic changes. For example, TNBC patients with negative methylation of the tumor suppressor gene *PCDH17* had significantly higher pCR rates [29]. Branhan et al. analyzed the methylation profile of TNBC tumors and defined hypomethylation as occurring in the DNA repair genes MMR. Interestingly, they found that genomic instability in TNBC is likely acquired via pathways other than the methylation of MMR genes [30]. Another study revealed that the hypomethylation of genes such as *CLEC14A* (cell adhesion), *MYO15B* (actin and ATP binding), *LTBR* (programmed cell death), *TMEM132D* and *TTC34* (transmembrane transporters and regulators) was the best predictive epigenetic marker for BC susceptibility to NACT [26]. The hypomethylation of two-pore domain potassium channel (*KCNK5* and *KCNK9*) was also observed, which correlated with their overexpression [31].

Consequently, among patients treated with NACT, those who experienced disease progression after chemotherapy had the highest levels of 5-mC and 5-hmC. Interestingly, the results of Pineda et al. showed that an algorithm using the methylation status of epigenetic signatures can accurately predict the response to chemotherapy with anthracyclines and taxanes. Higher methylation of the *FERD3L* (implicated in EMT) and *TRIP10* (implicated in cytoskeleton organization) genes in TNBC patients was correlated with better chemotherapy response, with an AUC = 0.905 (78.6% accuracy), whereas higher methylation of the *LOC641519*, *LEF1*, *HOXA5*, *EVC2*, *TLX3*, and *CDKL2* genes was identified in nonresponsive patients [32]. Meyer et al. identified nine differentially methylated regions that were significantly hypermethylated in nonresponder TNBC patients. Notably, all genes, such as *TMEM176A/B*, *UNC5D*, *STAC2*, *SDR42E1*, *NELL1*, *GRP*, *FOXG1*, *CDH8* and *GRIA4*, have been previously implicated in cancer and cancer-related pathways [33]. Furthermore, hypermethylation signatures have been linked to a shorter recurrence-free interval, regardless of whether patients received chemotherapy or not [34]. Notably, the role of the protein involved in the repair of DNA double-strand breaks (DSBs), such as BRCA, is also important. Up to 30% of TNBC cases have a BRCA mutation, which usually leads to a poorer prognosis. Interestingly, the lack of BRCA expression may result from the hypermethylation of the *BRCA1* promoter region [35,36] as well as hypomethylation of the ID4 repressor [30] and is associated with younger age, grade 3 tumors and a poor response to chemotherapy [35].

Our preliminary results shed light on the development of combination therapies with epidrugs to increase the efficacy of neoadjuvant chemotherapy. DNA methyltransferase inhibitors, such as azacitidine, decitabine, and guadecitabine, may restore aberrantly silenced genes and reprogram the epigenome, which may block breast cancer cell proliferation and/or sensitize tumors to other therapeutic interventions. However, early clinical trials have shown that DNA-methyltransferase inhibitors have limited efficacy as monotherapy in clinical trials of breast cancer [37]. On the basis of our observations, the combination of DNA methyltransferase inhibitors and neoadjuvant chemotherapy may enhance the therapeutic response in triple-negative breast cancer patients with elevated levels of DNA methylation markers. Notably, several in vitro studies support our hypothesis regarding the effectiveness of combining traditional chemotherapy with modern epidrugs [38,39,40].

Another interesting finding in our study was that the level of global methylation and demethylation of DNA, measured as the percentage of 5-mC and 5-hmC in DNA, was significantly correlated with the proliferation marker Ki-67 and tumor grade. High expression of Ki-67 has been significantly associated with more aggressive cancer features, such as lymph node metastasis, tumor invasion, high tumor nuclear grade, clinical stage, adverse survival outcomes, and failure to achieve a pathological complete response in TNBC patients [41]. Our results are inconsistent with those of Jin et al., who reported that proliferating cancer cells exhibiting high Ki-67 levels are often characterized by significantly reduced 5-hmC levels [42]. On the other hand, a correlation was observed between higher *DNMT1* expression and Ki-67 levels and higher histological grade in breast cancer, which partially supports our observation DNA hypermethylation [43,44]. Moreover, DNA methylation decreases *CREB3L1* mRNA expression, which is associated with increased tumor stage and shortened progression-free survival in patients with TNBC [45]. DNA methylation contributes to the downregulation of breast cancer metastasis suppressor gene 1 (*BRMS1*), while demethylation has been shown to inhibit the invasion of breast cancer cells [46].

In general, global DNA hypomethylation (reduced levels of 5-mC and 5-hmC) is frequently observed in various cancers, including breast cancer, and is associated with genomic instability and tumor progression [47,48]. Considering our observations, global hypomethylation in TNBC tissues seems to be a positive prognostic marker, as our results showed that individuals with the highest levels of global 5-mC and 5-hmC DNA were associated with poorer response to NACT, higher Ki-67 proliferation levels, and higher histological grade in triple-negative breast cancer tissues. This implies that higher levels of methylation could be linked to more aggressive and proliferative tumor characteristics.

Finally, some limitations and perspectives of this study are to be discussed. Unfortunately, one of the limitations of our work is the observation of methylation in tumors only at one time point—before the use of NACT. Repeated measurements before and after NACT could provide insights into the dynamic nature of DNA methylation patterns in response to treatment and its potential as a prognostic biomarker for breast cancer survival after neoadjuvant chemotherapy. Another shortcoming of this study is the method used for epigenetic analysis. ELISA-based assays are typically prone to high variability; thus, they are only suitable for the rough estimation of whole-genome methylation profiles. However, this is a simple and quick method that is effective for recognizing large alterations to the global DNA methylome, which can potentially be used as a rapid screening test for the initial assessment of the prediction of response to neoadjuvant chemotherapy. Obviously, our sample size was small as we challenged difficulties in collecting sufficient number of TNBC samples due to relatively low prevalence of this breast cancer subtype. A small sample size in epidemiological studies undermines the reliability and robustness of statistical analyses, making it more difficult to detect true effects, accurately estimate relationships, and generalize findings to the broader population. Thus, results of our study require further investigation. Also, it should be taken into account that this study involved two types of biological material: biopsies and surgical samples. Biopsy specimens, which were collected from NACT patients, may not be as tumor representative as surgical samples, since they are limited in size and may be taken from specific tumor regions like hypoxic areas or perinecrotic zones. To overcome this sampling bias, it would be more relevant to utilize liquid biopsies, which more accurately reflect tumor heterogeneity. Nevertheless, our findings underscore the potential of global methylation patterns as a valuable tool for predicting clinical outcomes in TNBC, prompting further research in this area. To overcome all the study limitations, future studies should utilize sufficient number of liquid biopsies in a comparative analysis of paired pre- and post-treatment samples.

Overall, the results of our study revealed that higher levels of 5-mC and 5-hmC markers were associated with poorer response to neoadjuvant chemotherapy, higher Ki-67 proliferation levels, and higher tumor grade in triple-negative breast cancer tissues. These observations suggest that hypermethylation could serve as a marker to identify patients with triple-negative breast cancer who are more likely to have a poorer response to chemotherapy and a more aggressive disease course. Understanding these correlations is crucial for developing personalized treatment plans and improving patient prognoses, emphasizing the need for further research into the methylation patterns in TNBC tissues and their impact on cancer proliferation markers such as Ki-67.

## 5. Conclusions

A significant correlation between the levels of 5-mC and 5-hmC in tumor tissue and prognostic markers such as Ki-67 and tumor grade suggests that markers of global DNA methylation and demethylation may also have prognostic significance in TNBC. More interestingly, the levels of 5-mC and 5-hmC in tumor tissue appear to be associated with the response to neoadjuvant therapy in patients with TNBC, suggesting that these epigenetic modifications may also have predictive value in treatment outcomes. If confirmed in larger cohorts, these findings could contribute to a better understanding of TNBC biology and aid in the development of personalized treatment strategies.

However, to fully establish the prognostic and predictive significance of these markers, further studies involving larger and more diverse patient cohorts are necessary. Future research should also focus on elucidating the molecular mechanisms underlying these epigenetic changes and their role in TNBC progression and therapy resistance. A deeper understanding of these processes may open new avenues for targeted therapeutic interventions in TNBC, a subtype that currently lacks effective molecularly targeted treatments.

## Figures and Tables

**Table 1:** Study group characteristics.

Group Characteristics	All *n* (%) Median (Min–Max)	NACT *n* (%) Median (Min–Max)	Without NACT *n* (%) Median (Min–Max)
Age [years]	53 (100%)	19 (100%)	34 (100%)
	57.2 (30–88)	49.0 (30–75)	60.4 (37–88)
Hormonal status			
ER-/PR/HER2-	53 (100%)	19 (100%)	34 (100%)
Ki-67	52 (98.1%)	18 (94.7%)	34 (100%)
	60 (8–90)	60 (25–90)	60 (8–90)
Tumor location			
Right	24 (45.3%)	11 (57.9%)	13 (38.2%)
Left	29 (54.7%)	8 (42.1%)	21 (61.8%)
DCIS			
Yes	18 (34.0%)	4 (21.1%)	14 (41.2%)
No	35 (66.0%)	15 (78.9%)	20 (58.8%)
G			
G1	1 (1.9%)	0 (0.0%)	1 (2.9%)
G2	24 (45.3%)	11 (57.9%)	13 (38.2%)
G3	28 (52.8%)	8 (42.1%)	20 (58.8%)
T			
cT1		1 (5.3%)	
cT2		11 (57.9%)	
cT3		5 (26.3%)	
cT4		2 (10.5%)	
			
ypT0		4 (21.1%)	
ypT1		7 (36.8%)	
ypT2		5 (26.3%)	
ypT3		2 (10.5%)	
ypT4		1 (5.3%)	
			
pT1			11 (32.3%)
pT2			19 (55.9%)
pT3			2 (5.9%)
pT4			2 (5.9%)
N			
cN0		7 (36.8%)	
cN1		7 (36.8%)	
cN2		3 (15.8%)	
cN3		2 (10.5%)	
			
ypN0		10 (52.6%)	
ypN1		6 (31.6%)	
ypN2		2 (10.5%)	
ypN3		1 (5.3%)	
			
pN0			21 (61.8%)
pN1			10 (29.4%)
pN2			2 (5.9%)
pN3			1 (2.9%)
M			
0	51 (96.2%)	18 (94.7%)	33 (97.1%)
1	2 (3.8%)	1 (5.3%)	1 (2.9%)
Stage			
IA	8 (15.1%)	0 (0.0%)	8 (23.5%)
IIA	21 (39.6%)	5 (26.3%)	16 (47.1%)
IIB	6 (11.3%)	4 (21.1%)	2 (5.9%)
IIIA	11 (20.8%)	6 (31.6%)	5 (14.7%)
IIIB	4 (7.5%)	2 (10.5%)	2 (5.9%)
IIIC	3 (5.7%)	2 (10.5%)	1 (2.9%)
			
Stage after NACT			
0		4 (21.1%)	
IA		4 (21.1%)	
IB		1 (5.3%)	
IIA		4 (21.1%)	
IIB		2 (10.5%)	
IIIA		1 (5.3%)	
IIIB		1 (5.3%)	
IIIC		1 (5.3%)	
IV		1 (5.3%)	
Surgery			
Mastectomy	29 (54.7%)	11 (57.9%)	18 (52.9%)
BCT	24 (45.3%)	8 (42.1%)	16 (47.1%)
Chemotherapy response			
pCR		4 (21.1%)	
partial response		7 (36.8%)	
stable disease		6 (31.6%)	
progression		2 (10.5%)	

**Table 2:** Markers of DNA methylation (5-mC) and DNA demethylation (5-hmC) in patients with triple-negative breast cancer with respect to the type of biological material.

Marker	All Samples (*n* = 53)	Biopsies from Patients with NACT (*n* = 19)	Surgical Samples from Patients Without NACT (*n* = 34)	*p* *
5-mC [%]	2.129 ± 2.005 1.467 (0.009–7.937)	0.570 ± 0.826 0.176 (0.008–3.237)	3.000 ± 1.944 2.859 (0.010–7.937)	<0.0001
5-hmC [%]	0.166 ± 0.175 0.106 (0.010–0.849)	0.060 ± 0.072 0.026 (0.010–0.263)	0.225 ± 0.189 0.239 (0.012–0.849)	<0.001

5-mC—5-methylcytosine; 5-hmC—5-hydroxymethylcytosine; NACT—neoadjuvant chemotherapy. Data are presented as the means with SDs (standard deviations) and medians with ranges. *—Group differences were analyzed with the Mann–Whitney U test.

**Table 3:** Pretreatment levels of markers of DNA methylation (5-mC) and DNA demethylation (5-hmC) in patients with triple-negative breast cancer with respect to the response to neoadjuvant chemotherapy.

Marker	Response to NACT				
	pCR (*n* = 4)	Partial Response (*n* = 7)	Stable Disease (*n* = 6)	Progression (*n* = 2)	*p* *
5-mC [%]	0.095 (0.008–0.293)	0.011 (0.010–1.441)	0.582 (0.012–0.893)	2.290 (1.343–3.237)	0.129
5-hmC [%]	0.019 (0.010–0.078)	0.034 (0.010–0.127)	0.013 (0.010–0.077)	0.232 (0.200–0.263)	0.080

5-mC—5-methylcytosine; 5-hmC—5-hydroxymethylcytosine; NACT—neoadjuvant chemotherapy; pCR—pathological complete response. Data are expressed as median values with ranges. *—Group differences were analyzed with the Kruskal–Wallis test.

## Data Availability

The original contributions presented in this study are included in the article and Supplementary Materials. Further inquiries can be directed to the corresponding author.

## Supplementary Materials

The following supporting information can be downloaded at: https://www.mdpi.com/article/10.3390/biomedicines13030585/s1, Figure S1: Correlation between patients’ age and epigenetic markers; Figure S2: Pretreatment levels of epigenetic markers in patients with NACT; Figure S3: Levels of epigenetic markers in patients without NACT.

## Abbreviations

The following abbreviations are used in this manuscript:

5-hmC	5-hydroxymethylcytosine
5-mC	5-methylcytosine
ALDH1	Aldehyde dehydrogenase 1 family
AUC	area under the curve
BC	breast cancer
Bcl2	BCL2 apoptosis regulator
BRMS1	breast cancer metastasis suppressor gene 1
BSA	bovine serum albumin
c	clinical stage
CD4	CD4 T lymphocytes
CD8+	cytotoxic T cells
DCIS	ductal carcinoma in situ
DNMT	DNA methyltransferases
DSBs	DNA double-strand breaks
EMT	epithelial–mesenchymal transition
ER	estrogen receptor
FFPE	formalin-fixed, paraffin-embedded
FGFR4	fibroblast growth factor receptor 4
HAGE	helicase antigen gene
HER2	human epidermal growth factor receptor 2
HRP	horseradish peroxidase
Ki-67	antigen kiel 67
MMP7	matrix metallopeptidase 7
NACT	neoadjuvant chemotherapy
NST	no special type
NUP98	nucleoporin 98
p	pathological stage
pCR	pathological complete response
PD-L1	programmed cell death ligand 1
PR	progesterone receptors
RCB	residual cancer burden
TET	ten-eleven translocation
TILs	tumor-infiltrating lymphocytes
TNBC	triple-negative breast cancer
TNM	tumor, nodes, metastasis
TOPK	T-LAK cell-originated protein kinase
VEGFR2	vascular endothelial growth factor receptor 2
YAP1	Yes1 associated transcriptional regulator
yp	post-treatment pathological stage
