# Supplementary material for: DNA Methylation and Demethylation in Triple-Negative Breast Cancer: Associations with Clinicopathological Characteristics and the Chemotherapy Response
Source: Biomedicines. 2025 Feb 26;13(3):585. doi: 10.3390/biomedicines13030585 (PMC11939961; doi:10.3390/biomedicines13030585)
Supplement: Supplementary file 1 [file biomedicines-13-00585-s001.zip › biomedicines-3467333-supplementary.pdf]

Supplementary

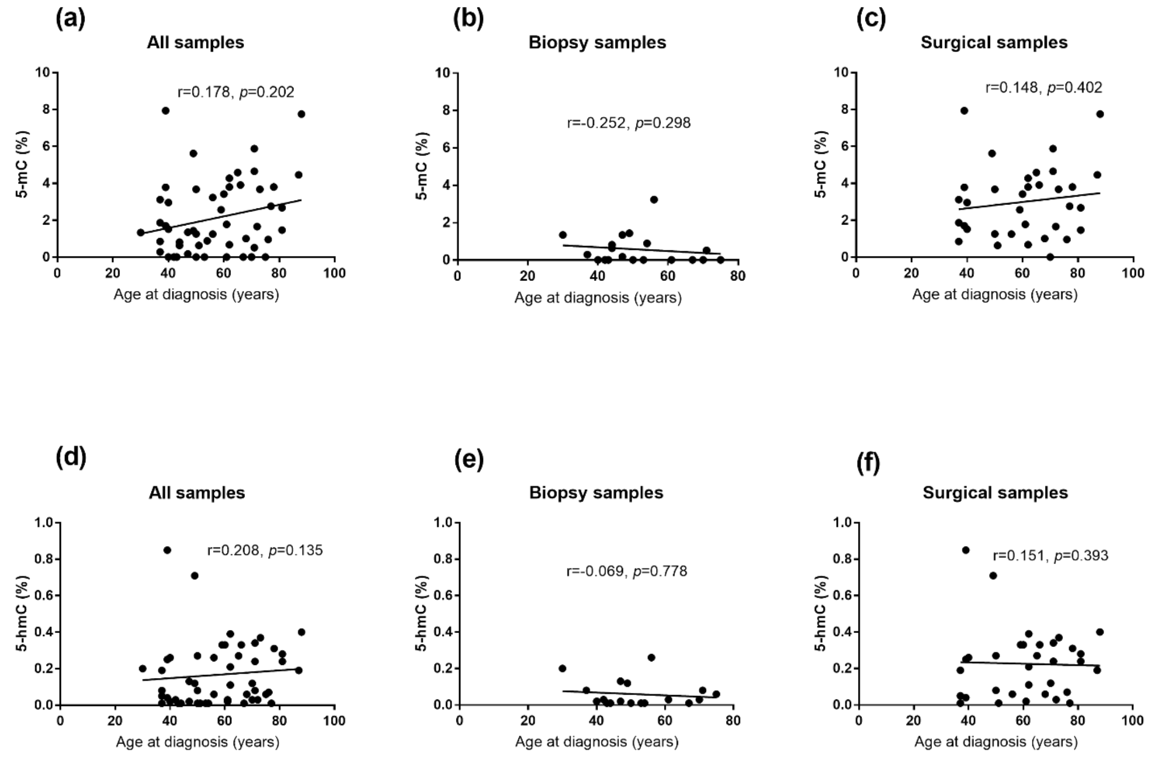

Figure S1. **Correlation between patients' age and epigenetic markers.** No correlation between patients' age and markers of DNA methylation/demethylation (5-mC/5-hmC) in the collected tissues, including samples from all TNBC patients (a, d), biopsies collected from TNBC patients before neoadjuvant chemotherapy (b, e) and surgical samples from TNBC patients not treated with neoadjuvant chemotherapy (c, f).

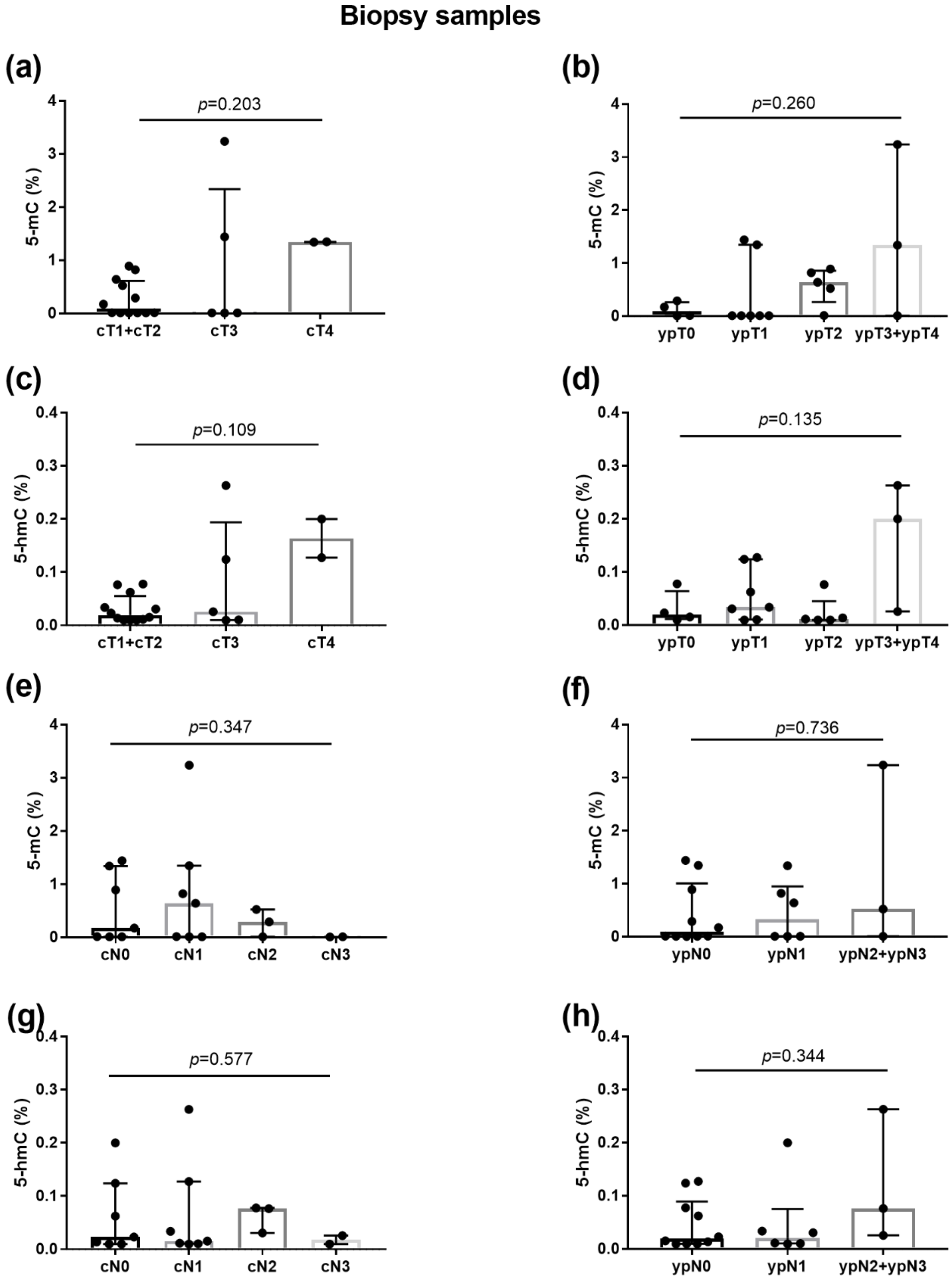

Figure S2. Pretreatment levels of epigenetic markers in patients with NACT. Pretreatment levels of markers of DNA methylation/demethylation (5-mC/5-hmC), measured in biopsies collected from patients undergoing neoadjuvant chemotherapy. Data stratified by clinical tumor stage, cT (a/c) and cN (e/g), and posttreatment pathological tumor stage, ypT (b/d) and ypN (f/h). Because of limited sample sizes in ypT4 and ypN3 (with only one observation in each), these groups were merged, with ypT3 and ypN2, respectively. Group differences were analyzed with the Kruskal–Wallis test. Data are shown as raw values and medians with interquartile ranges.

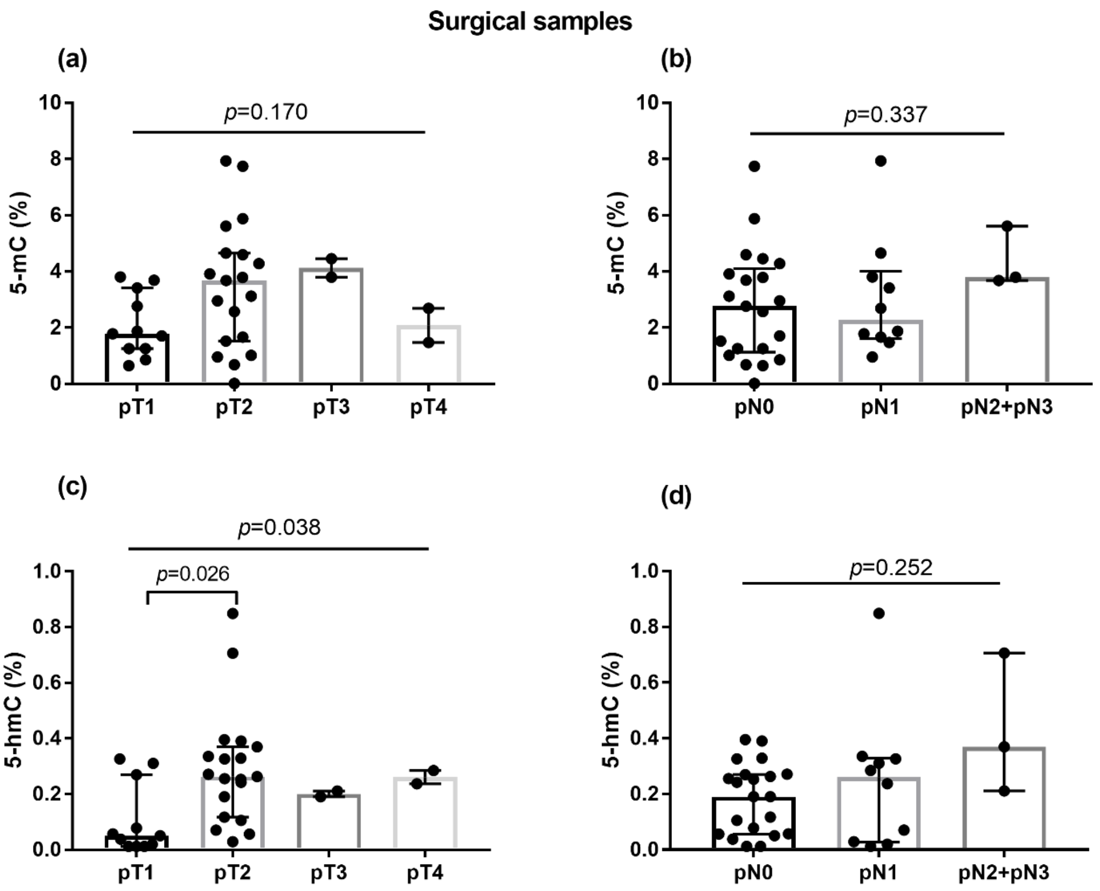

Figure S3. Levels of epigenetic markers in patients without NACT. Levels of markers of DNA methylation/demethylation (5-mC/5-hmC), measured in surgical samples collected from TNBC patients not treated with neoadjuvant chemotherapy. Data stratified by pathological tumor stage: pT (a/c) and pN (b/d). Because of the limited sample size in pN3 (only one observation), this group was merged with pN2. Group differences were analyzed with the Kruskal–Wallis test followed by Dunn's test. Data are shown as raw values and medians with interquartile ranges.
